# Risk factors for intraoperative in-stent thrombosis during stent-assisted coiling of paraclinoid aneurysms

**DOI:** 10.3389/fneur.2023.1333075

**Published:** 2024-01-12

**Authors:** Chun Zeng, Jing Wang

**Affiliations:** ^1^Department of Neurosurgery, Beijing Tiantan Hospital, Capital Medical University, Beijing, China; ^2^China National Clinical Research Center for Neurological Diseases, Beijing, China; ^3^Department of Neurosurgery, Peking University International Hospital, Beijing, China

**Keywords:** intracranial aneurysm, paraclinoid aneurysm, in-stent thrombosis, thromboelastography, morphology, risk factors, stent-assisted coiling

## Abstract

**Objectives:**

To identify independent risk factors for intraoperative in-stent thrombosis (IST) in paraclinoid aneurysms (PAs).

**Methods:**

172 PA patents undergoing stent-assisted coiling (SAC) were divided into an IST group (*n* = 12) and a non-IST group (*n* = 160). Clinical characteristics, aneurysm morphologies, and laboratory parameters were measured. We performed independent *t* tests (for normally distributed data) or non-parametric tests (for non-normally distributed data) to compare continuous parameters. Multivariate logistic regression analysis with a stepwise forward method was conducted to determine independent risk factors. Receiver operating characteristic curves were generated, and the Delong test was employed for comparisons.

**Results:**

Independent risk factors for IST included size ratio (SR) (*p* < 0.001, odds ratio [OR] = 3.909, confidence interval [CI] = 1.925–7.939), adenosine diphosphate (ADP) inhibition (*p* = 0.028, OR = 0.967, CI = 0.938–0.996), and reaction time (R) (*p* = 0.006, OR = 0.326, CI = 0.147–0.725). The combined factors (SR, ADP inhibition, and R) exhibited area under the curves of 0.870, 0.720, 0.716, and 0.697, with cutoff values of 2.46, 69.90%, and 4.65, respectively.

**Conclusion:**

The SR, ADP inhibition, and *R* values were independent risk factors for the IST in the PAs undergoing SAC. For PAs with a large SR, surgeons could prepare for long-term dual antiplatelet therapy before SAC.

## Introduction

Intracranial aneurysms (IAs) pose significant health risks, and their incidence has increased with the development of non-invasive imaging techniques (1, 2). Ruptured aneurysms occur in 8.0 per 100,000 persons per year, while many aneurysms, particularly paraclinoid aneurysms (PAs), remain unruptured (2, 3). PAs arise from the distal dural ring of the internal carotid artery to the initial segment of the posterior communicating artery (4). Despite the low rupture risk, PAs can grow and impinge on adjacent structures, causing symptoms such as headaches, visual field defects, eye movement abnormalities, and changes in pupil size (5). Despite the widespread use of flow diverters (FDs) for internal carotid artery aneurysms, stent-assisted coiling (SAC) remains a valuable option for treating of PAs, given its lower complication rates and cost-effectiveness (6, 7). However, intraoperative in-stent thrombosis (IST) poses a significant risk, even with routine dual antiplatelet therapy (DAPT) (8). Identifying risk factors for IST in PAs before surgery is crucial.

Prior research has focused on factors like stent manipulation, parent artery characteristics, hyperlipidemia, stent type, and operation duration as primary risk factors for IST in PAs (8, 9). Thromboelastography (TEG) has been used clinically to monitor platelet aggregation, with adenosine diphosphate (ADP) inhibition and arachidonic acid (AA) inhibition gaining attentions. Furthermore, few studies have explored the impact of aneurysm morphologies on the development of IST. The coil embolization process can influence local blood flow from the aneurysm dome to the neck, and unstable flow can increase the risk of localized hypercoagulation (10). The complexity of the coiling procedure and extended operation time, particularly when employing the Jailing technique, is associated with various aneurysm morphologies. Thus, our analysis encompassed morphological parameters, clinical characteristics, and laboratory parameters to identify risk factors for IST in PAs during SAC.

## Methods

### Patients and data

**Figure 1:**
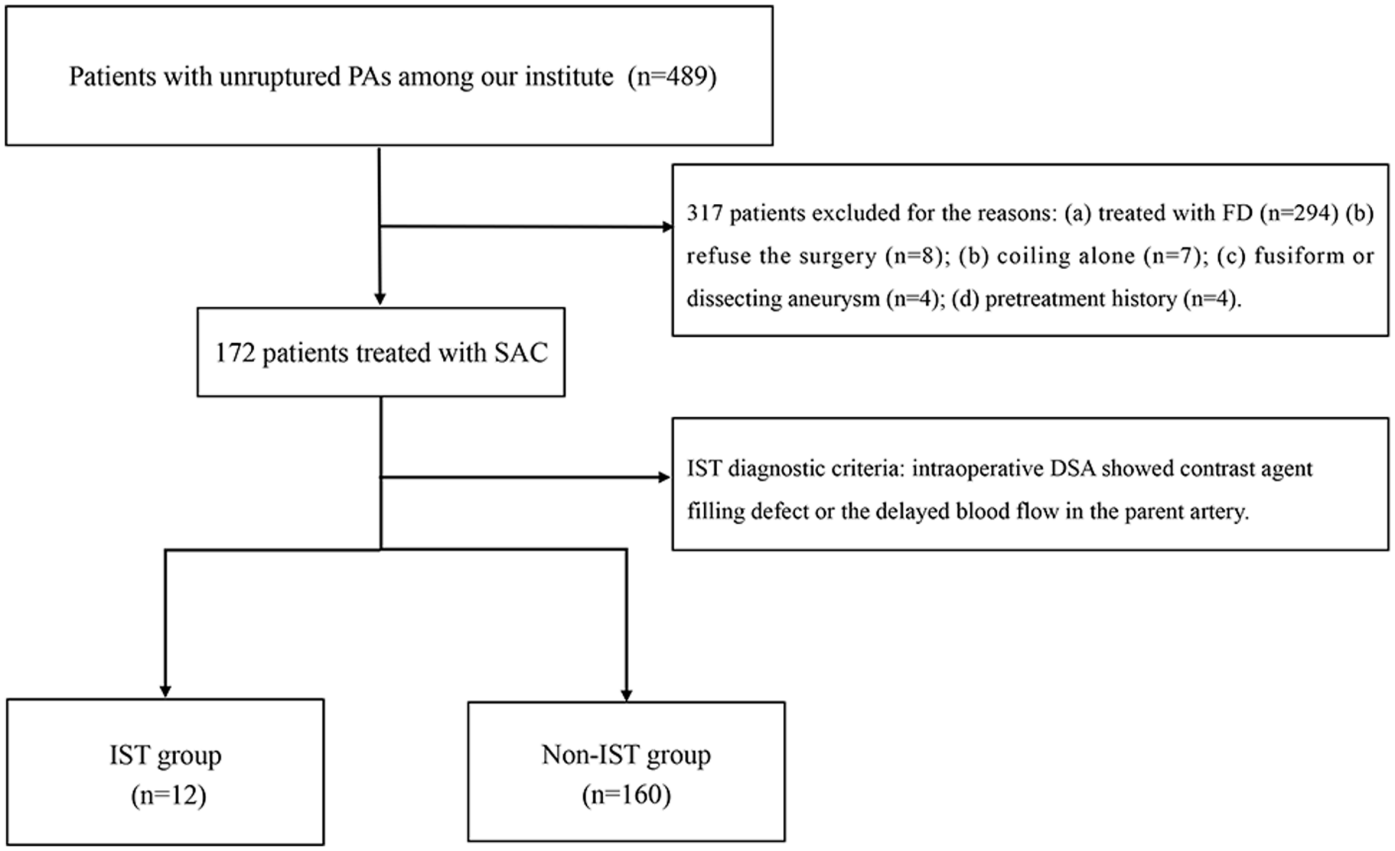
The flow chart of patient selection. PAs, Paraclinoid aneurysms; DSA, Digital subtraction angiography; FD, Flow diverter; and IST, In-stent thrombosis.

**Figure 2:**
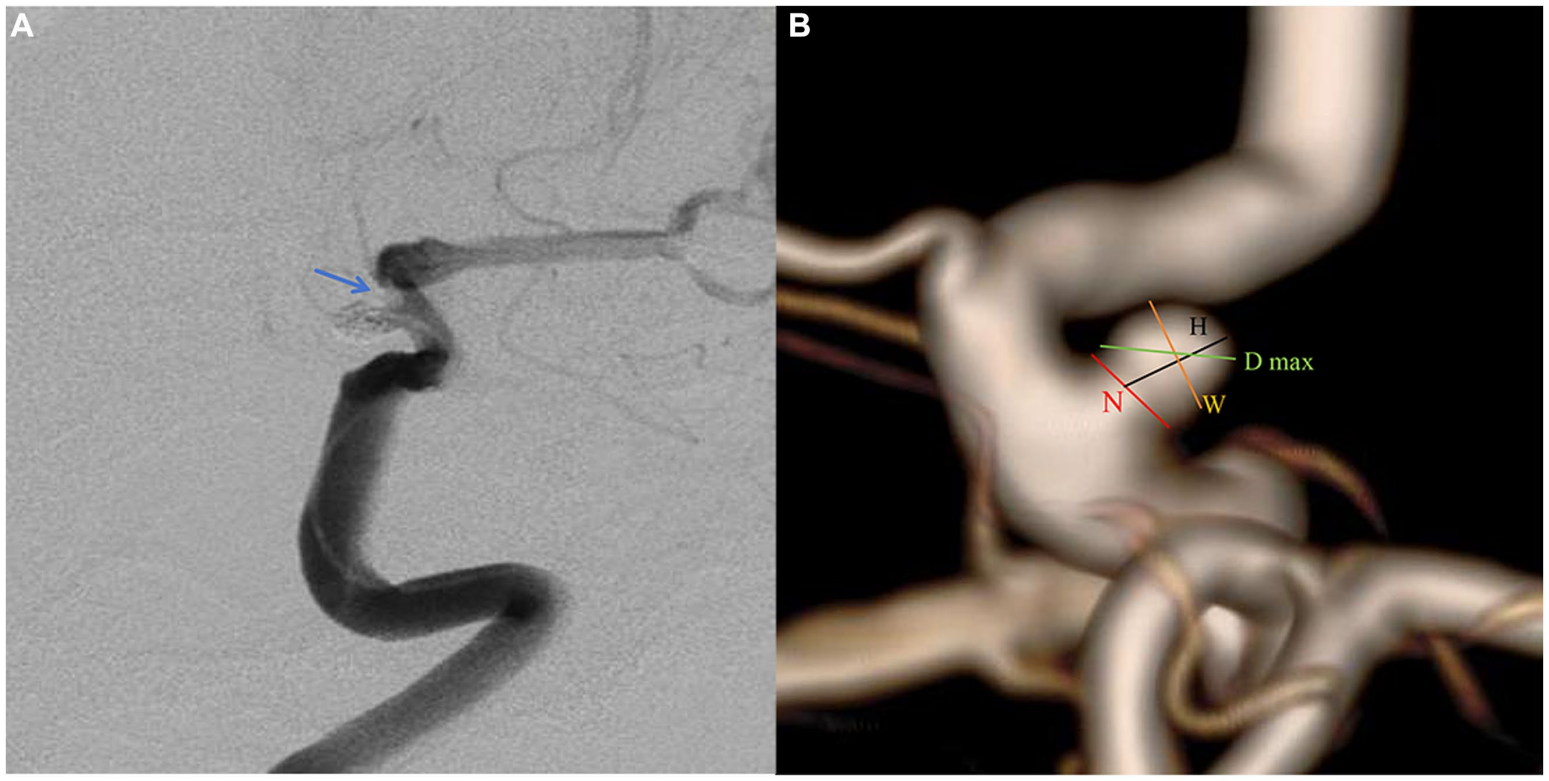
A paraclniod aneurysm of two-dimension and three-dimension digital subtraction angiography (DSA). **(A)** Two-dimension DSA showing the in-stent thrombosis (blue arrow). **(B)** The definition of morphologic variables in three-dimension DSA.

The patient selection flowchart is presented in Figure 1. The study received approval from our hospital’s institutional ethics committee, and we collected data after obtaining informed consent from the patients or their close relatives. From May 2021 to September 2022, 489 patients with unruptured PAs via digital subtraction angiography (DSA), and 172 underwent SAC. 317 patients were excluded for the following reasons: (a) FD treatment (*n* = 294); (b) refusal to undergo surgery (*n* = 8); (b) coiling alone (*n* = 7); (c) fusiform or dissecting aneurysm (*n* = 4); and (d) prior treatment history (*n* = 4). The 172 patients were divided into an IST group (*n* = 12) and a non-IST group (*n* = 160). The diagnostic criteria for IST included intraoperative DSA demonstrating contrast agent filling defects or delayed blood flow in the parent artery (Figure 2A).

We recorded clinical characteristics [age and gender, smoking, alcohol, hypertension, diabetes mellitus (DM), hyperlipemia, and coronary heart disease], operation data [aneurysm side, stent type (laser-cut or braided stents), and operation time], aneurysm morphology, and laboratory test results.

### Procedure

All patients underwent endovascular treatment for PAs. Intraoperative heparinization and a preoperative combination of aspirin (100 mg/day) and clopidogrel (75 mg/day; or ticagrelor 90 mg twice daily) were administered to PAs patients for 3–5 days, AA inhibition exceeded 50%, and ADP inhibition exceeded 30%. The coiling microcatheter was initially positioned at the aneurysm’s base, followed by the deployment of a stent at the aneurysm neck. This stent expansion effectively secured the coiling microcatheter against the parent artery’s wall. DSA was performed before each coil detachment and at the procedure’s conclusion to assess parent vessel patency and confirm all coils were confined to the target aneurysm. In cases of IST, tirofiban treatment was administered, either intravenously at 8–10 μg/kg for 3 min or through targeted arterial infusion at 0.5–1.0 mg at a rate of 1–5 mL/min. If no thrombus dissolution signs were observed, these doses were readministered until intraoperative angiography confirmed thrombus dissolution and blood flow restoration. Subsequently, intravenous administration at 0.1–0.5 μg·kg^-1^·min^-1^ continued for 24–48 h post-procedure. No systematic complications were reported in the IST group following tirofiban treatment. For the subsequent 3 months, patients received daily doses of 75 mg clopidogrel or 90 mg ticagrelor, along with 100 mg aspirin daily for 6 months.

### Morphologic parameter measurement

Pretreatment DSA images and three-dimensional reconstructions were obtained for each patient using the Innova Workplace system (GE Medical). Two experienced neuro-interventional surgeons performed measurements of morphological parameters using the same procedure, and we defined the parameters as the average of their values. The results were precise to two decimal places. The morphologic variables were defined as follows (Figure 2B):

1. Maximum diameter (D max): The aneurysm’s maximum diameter.
2. Height (H): The maximum distance from the center of the aneurysm neck to a point on the sac.
3. Width (W): The maximum distance perpendicular to H.
4. Neck diameter (N): The maximum diameter in the neck plane.
5. Volume (V): Aneurysm lumen volume calculated in 3D space.
6. Neck space (S): The area of the projection plane of the neck surface.
7. Parent artery diameter (PD): The average value of vessel diameters at the proximal and distal sites.
8. Bottleneck factor (BNF): The ratio of width to neck width.
9. H/W: The ratio of H to W.
10. Size ratio (SR): The ratio of H to the Parent artery diameter.
11. Aspect ratio (AR): The ratio of H to N.
12. Volume neck space ratio (VNR): The ratio of V to S.

### Laboratory tests

The laboratory parameters analyzed in this study included red blood cell counts, hemoglobin levels, white blood cell counts, platelet counts, cholesterol levels, triglyceride levels, blood glucose levels, prothrombin time, prothrombin time activity, international normalized ratio, fibrinogen levels, activated partial thromboplastin time, thrombin time, AA inhibition, ADP inhibition, reaction time (R), kinetics time, Alpha angle, maximum amplitude, estimated percent lysis, coagulation index, and Lysis 30.

### Statistical analysis

Statistical analysis was performed using SPSS 20.0 (IBM Inc., Chicago, IL, United States). The normality of the data was determined using the Shapiro–Wilk test. Continuous variables with a normal distribution were expressed as mean ± standard deviation, while those without a normal distribution were expressed as median ± interquartile range. Categorical variables were reported as frequencies (percentages). An independent *t* test (for normally distributed data) or non-parametric test (for non-normally distributed data) was used for continuous variables, and the chi-square test was used for categorical variables. Variables that were significant in the univariate analysis (operation duration, D max, H, W, H/W, V, SR, AR, VNR, ADP inhibition, and R) were included in a multivariate logistic regression analysis using the stepwise forward method to identify independent risk factors. Receiver operating characteristic curves were generated to calculate the area under the curve (AUC) and cutoffs for independent variables. The AUCs were compared using the DeLong test. Differences were considered statistically significant when *p* < 0.05.

## Results

### Clinical characteristics

Out of the 172 patients who underwent SAC, 12 (6.98%) experienced IST. The average age of the patients was 53.40 years, with 37 (21.51%) males, 12 (6.98%) smokers, 15 (8.72%) drinkers, 49 (28.49%) having hypertension, eight (4.65%) with DM, 36 (20.93%) with hyperlipemia, and five (2.91%) with coronary heart disease. One hundred (58.14%) of the PAs were located on the left. Of the 172 stents used, 131 (76.16%) were Enterprise, 32 (18.60%) were Lvis, five (2.91%) were Neuroform EZ, and four (2.33%) were Solitare. These stents were classified as either laser-cut or braided stents.

Table 1 shows the clinical characteristics and operation data of the IST and non-IST groups. There were no significant differences in age, gender, aneurysm side, smoking, alcohol, hypertension, DM, hyperlipemia, coronary heart disease, or stent type (*p* > 0.05). However, the IST group had a significantly longer operation time compared to the non-IST group (113.58 vs. 70.00 min, *p* < 0.001).

**Table 1:** Univariate analysis of clinical characteristics, morphologies, and laboratory parameters.

Variables	IST group (*n* = 12)	Non-IST group (*n* = 160)	*p* value
Clinical characteristics			
Age, year	54.92 ± 11.33	53.29 ± 9.08	0.557^a^
Gender			0.249^c^
Male	1 (8.33%)	36 (22.50%)	
Female	11 (91.67%)	124 (77.50%)	
Aneurysm side			0.535^c^
Left	8 (66.67%)	92 (57.50%)	
Right	4 (33.33%)	68 (42.50%)	
Smoking	1 (8.33%)	11 (6.88%)	0.848^c^
Alcohol	1 (8.33%)	14 (8.75)	0.961^c^
Hypertension	6 (50.00%)	43 (26.88%)	0.087^c^
Diabetes mellitus	0 (0.00%)	8 (5.00%)	0.428^c^
Hyperlipemia	4 (33.33%)	32 (20.00%)	0.273^c^
Coronary heart disease	1 (8.33%)	4 (2.50%)	0.246^c^
Stent type			0.174^c^
Laser-cut	8 (66.67%)	132 (82.50%)	
Braided	4 (33.33%)	28 (17.50%)	
Operation time, min	113.58 ± 33.49	70.00 ± 33.75	**< 0.001** ^**b**^
Morphology			
D max, mm	9.59 ± 4.14	5.84 ± 3.05	**0.004** ^**b**^
H, mm	5.47 ± 6.22	3.97 ± 2.36	**0.001** ^**b**^
W, mm	7.52 ± 3.20	4.83 ± 2.13	**0.013** ^**b**^
H/W	1.01 ± 0.23	0.81 ± 0.23	**0.014** ^**b**^
V, mm^3^	114.74 ± 462.81	40.38 ± 69.13	**0.006** ^**b**^
S, mm^2^	24.00 ± 40.69	19.60 ± 17.42	0.060^b^
N, mm	5.53 ± 3.73	5.00 ± 2.22	0.060^b^
PD, mm	3.87 ± 0.90	4.02 ± 0.66	0.218^b^
BNF	1.01 ± 0.32	0.99 ± 0.19	0.560^b^
SR	1.64 ± 1.89	1.02 ± 0.72	**0.011** ^**b**^
AR	1.01 ± 0.39	0.79 ± 0.30	**0.009** ^**b**^
VNR	5.25 ± 5.54	2.63 ± 2.29	**0.018** ^**b**^
Laboratory test			
Red blood cell	4.11 ± 0.44	4.24 ± 0.44	0.326^a^
Hemoglobin	127.5 ± 11.75	126.93 ± 14.17	0.942^b^
White blood cell	5.72 ± 1.48	5.25 ± 1.64	0.339^b^
Platelet	212.17 ± 47.98	204.00 ± 61.75	0.831^b^
Cholesterol	4.20 ± 0.97	4.23 ± 1.40	0.878^b^
Triglyceride	1.28 ± 0.53	1.17 ± 0.77	0.692^b^
Blood Glucose	5.13 ± 0.57	4.94 ± 0.81	0.705^b^
Prothrombin time	12.94 ± 0.49	13.03 ± 0.55	0.590^a^
Prothrombin time activity	105.42 ± 9.87	102.00 ± 14.00	0.364^b^
International normalized ratio	0.98 ± 0.05	0.99 ± 0.08	0.361^b^
Fibrinogen	2.97 ± 0.78	2.85 ± 0.73	0.489^b^
Activated partial thromboplastin time	36.70 ± 9.68	36.85 ± 4.68	0.641^b^
Thrombin time	16.88 ± 0.69	17.10 ± 1.25	0.514^b^
AA inhibition	100.00 ± 2.10	100.00 ± 4.20	0.605^b^
ADP inhibition	46.59 ± 12.74	65.10 ± 41.88	**0.012** ^**b**^
Reaction time	4.81 ± 1.10	5.35 ± 1.40	**0.023** ^**b**^
kinetics time	1.33 ± 0.45	1.30 ± 0.50	0.436^b^
Alpha angle	75.95 ± 6.33	74.05 ± 8.15	0.148^b^
Maximum amplitude	63.18 ± 4.04	62.10 ± 5.95	0.409^b^
Estimated percent lysis	0.00 ± 0.00	1.35 ± 1.90	0.633^b^
Coagulation index	1.73 ± 1.40	1.35 ± 1.90	0.251^b^
Lysis 30 min	0.00 ± 0.00	0.00 ± 0.00	0.496^b^

*p* < 0.05 were in bold.

^a^ performed independent *t* test, ^b^ non-parametric test, ^c^ chi-square test.

IST, In-stent thrombosis; D max, Maximum diameter; W, Width; H, Height; V, Volume; S, Space; N, Neck diameter; PD, Parent artery diameter; BNF, Bottleneck factor; SR, Size ratio; AR, Aspect ratio; VNR, Volume neck space ratio; AA, Arachidonic acid; and ADP, Adenosine diphosphate.

### Morphologic and laboratory predictors

Table 1 displays the values of morphologic and laboratory parameters. Univariate analysis revealed that the IST group had significantly greater H (5.47 vs. 3.97 mm, *p* = 0.001), W (7.52 vs. 4.83 mm, *p* = 0.013), D max (9.59 vs. 5.84 mm, *p* = 0.004), H/W (1.01 vs. 0.81, *p* = 0.014), V (114.74 vs. 40.38 mm^3^, *p* = 0.006), SR (1.64 vs. 1.02, *p* = 0.011), AR (1.01 vs. 0.79, *p* = 0.009), and VNR (5.25 vs. 2.63, *p* = 0.018), compared to the non-IST group. However, there was no significant difference in S (24.00 vs. 19.60 mm^2^), N (5.53 vs. 5.00 mm), PD (3.87 vs. 4.02 mm), and BNF (1.01 vs. 0.99) (*p* > 0.05). The IST group had significantly lower ADP inhibition (46.59 vs. 65.10%, *p* = 0.012) and R value (4.81 vs. 5.35 min, *p* = 0.023) compared to the non-IST group.

**Figure 3:**
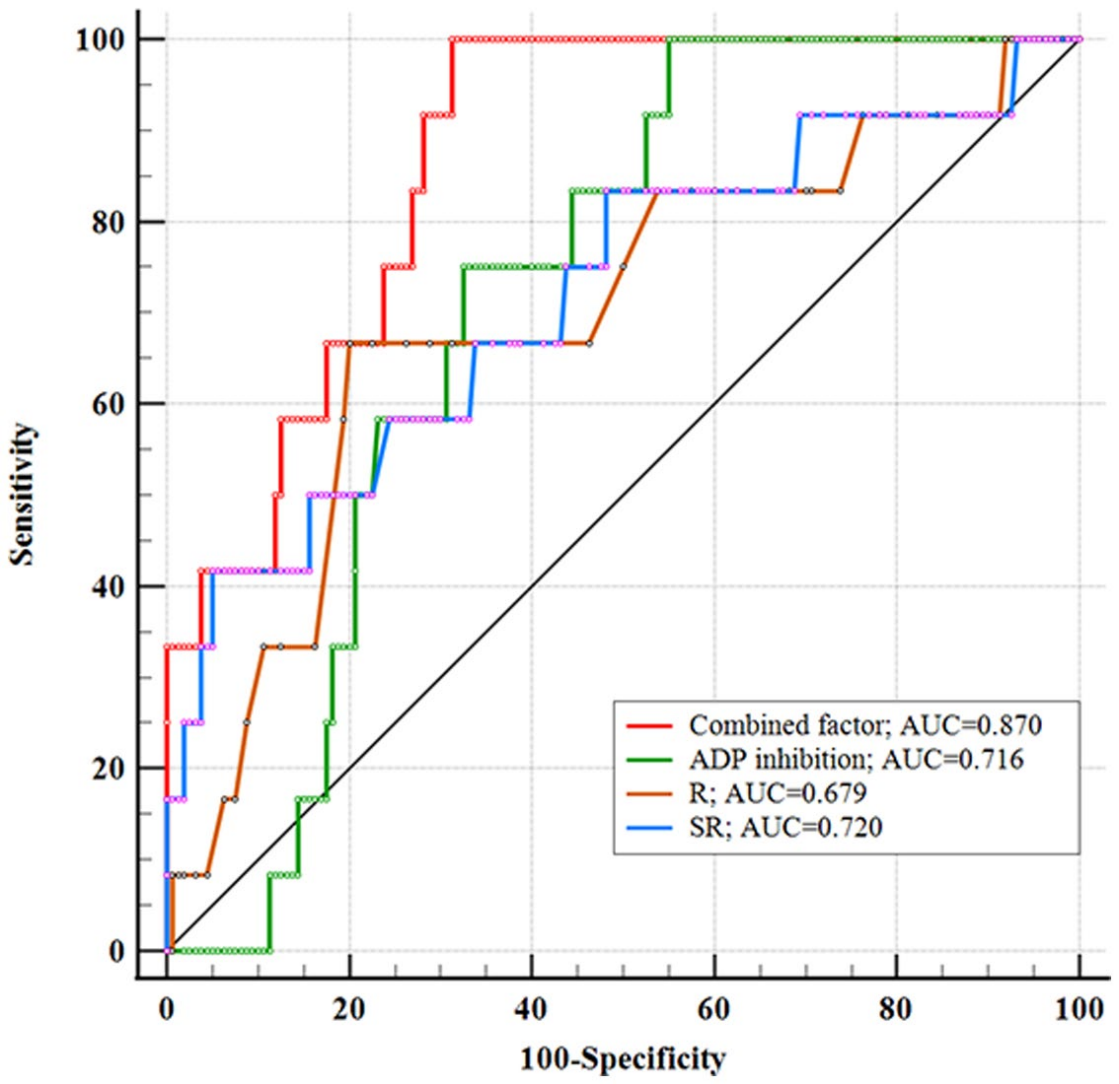
Receiver operating characteristic showing the area under the curve (AUC) of combined factor, SR, ADP inhibition, and R. The combined factor integrating SR, ADP inhibition, and R performed high performance.

We conducted multivariate logistic regression analysis using the stepwise forward method to identify independent predictors of IST (Table 2). SR (*p* < 0.001, OR = 3.909, CI = 1.925–7.939), ADP inhibition (*p* = 0.028, OR = 0.967, CI = 0.938–0.996), and R (*p* = 0.006, OR = 0.326, CI = 0.147–0.725) were determined as independent risk factors. AUCs of the combined factor (SR + ADP + R), SR, ADP inhibition, and R were 0.870, 0.720, 0.716, and 0.697, respectively. The AUC of the combined factor was significantly higher than the AUCs of SR and ADP inhibition (0.870 vs. 0.720, 0.797, respectively, *p* < 0.05, Delong test), but only insignificantly higher than R (0.870 vs. 0.697, *p* = 0.059, Delong test; Figure 3). The cutoff values for SR, ADP inhibition, and R were 2.46 mm, 69.90%, and 4.65 min, respectively.

**Table 2:** Multivariate logistic analysis results.

Variables	β value	*p* value	OR	95% CI
ADP inhibition	−0.034	0.028	0.967	0.938–0.996
SR	1.363	< 0.001	3.909	1.925–7.939
R	−1.120	0.006	0.326	0.147–0.725
Constant	2.685	0.194	14.655	

ADP, Adenosine diphosphate; SR, Size ratio; R, Reaction time; OR, Odds ratio; and CI, Confidence interval.

Furthermore, we categorized patients into a high SR cohort (*n* = 13) and a low SR cohort (*n* = 159) based on the cutoff value. The high SR cohort exhibited a significantly higher percentage of IST compared to the low SR cohort (38.46 vs. 4.40%, *p* = 0.001). Among the high SR group, 10 (76.92%) patients had ADP inhibition of less than 69.9% or R values under 4.65 min.

## Discussion

Stent-assisted coiling and FDs are the primary treatments for PAs, with stents being the unavoidable option for wide-necked PAs (6, 11). Although indications for FDs have expanded, some institutions prefer them for handling giant, multiple, tandem, dissecting, or recurrent aneurysms. Meanwhile, SAC remains a suitable option for single, small, or medium-sized anterior circulation aneurysms, given its technical maturity, lower complication risks, and cost-effectiveness (6, 12). Furthermore, FDs require long-term DAPT and more frequent angiography. A study involving 400 unruptured PAs highlighted ischemic complications as the most common, with an incidence of up to 15% (13). This could be attributed to factors such as stent histocompatibility, vascular intimal injury, stent mal-apposition, and inadequate DAPT effects (14, 15). Despite DAPT preparation, intraoperative in-stent thrombosis (IST) may occur, with aneurysm morphology potentially contributing—a factor that has received limited attention in other institutions. Preoperative analysis of what types of aneurysms are likely to cause IST situations will help prepare better. Therefore, we systematically analyzed the clinical characteristics, aneurysm morphology, and laboratory parameters of patients with unruptured PAs. The incidence of IST was 6.98%, aligning with the findings of Liu et al. (16). Significant independent predictors were more significant SR, insufficient ADP inhibition, and lower R. AUCs of the combined factor (SR + ADP + R), SR, ADP inhibition, and R were 0.870, 0.720, 0.716, and 0.697, respectively.

Thromboelastography emerges as a valuable tool for monitoring antiplatelet drug efficacy (17). Studies have indicated that approximately one-third of patients, particularly those on clopidogrel, are resistant to antiplatelet drugs, rendering them susceptible to ischemic events (17). Clopidogrel, acting via the ADP pathway, requires sufficient DAPT to prevent thrombosis events (18). Some studies have suggested genetic testing as an improvement and proposed replacing clopidogrel with ticagrelor (19, 20). Fuga et al. discovered that 43 (51%) patients who experienced ischemic events after SAC found ADP inhibition <28.8% to be associated with ischemia in unruptured IAs (17) This underscores that merely meeting DAPT criteria is insufficient for SAC. In our study, the cutoff value for ADP inhibition in PAs undergoing SAC was found to be over 69.90%, establishing a comparatively safe threshold. R signifies the latency period between initiating the coagulation system reaction and forming a fibrin clot. A low R value indicates hypercoagulability. A study of 261 unruptured aneurysms described patients with microbleeds and highlighted the R value, supporting the notion that a low R may increase coagulation (21).

While several morphologic parameters including H, W, H/W, AR, and V showed statistically significant differences in the univariate analysis, only SR was found to be an independent risk factor of IST in PAs. To our knowledge, no study has reported the association between SR and IST. SR represents the overall complexity of the aneurysm and parent artery. Aneurysms located on smaller arteries exhibit abnormal sac walls and unstable hemodynamics linked to the initiation, growth, and development of intracranial aneurysms (22). Larger PAs pose challenges during coiling, potentially requiring prolonged operation times, thus elevating the risk of IST due to repeated manipulation and intimal injury. This clarifies why operation time was significant in univariate analysis but not in multivariate analysis. Additionally, a smaller PD implies a narrower stent space and a heterogeneous parent artery, affecting stent release. This emphasizes the need for a comprehensive consideration of the status of intracranial aneurysms and the parent artery.

It is worth noting that while each individual predictor demonstrated good performance in predicting IST, combining all independent predictors resulted in even better accuracy (AUC = 0.870). Furthermore, the effectiveness of ADP inhibition and R value could be improved by prolonging the duration of DAPT. Our study also indicated that for PAs with large SR, an extended DAPT period is recommended to prevent IST during SAC.

This study has several limitations that should be considered. First, its retrospective design introduces a risk of bias and confounding factors. Second, the limited variety of stent types used in our study prevented a more comprehensive categorization. Third, a retrospective review of images may not have allowed for discernment of some stent apposition. Fourth, our sample size was limited due to the impact of COVID-19 and the controversial nature of active treatment. Our center recommended that stable PA patients be followed up as outpatients. Finally, this study only focused on PAs and did not include multiple locations. Therefore, future multicenter prospective studies are necessary to identify additional risk factors for IST.

## Conclusion

The SR, ADP inhibition, and R values were identified as independent risk factors for the IST in PAs undergoing SAC. For PAs with a large SR, surgeons could consider an extended DAPT period before SAC.

## Data availability statement

The raw data supporting the conclusions of this article will be made available by the authors, without undue reservation.

## Ethics statement

The studies involving humans were approved by the Ethics Committee of Beijing Tiantan Hospital (Approval No. KY 2023–261-01). The studies were conducted in accordance with the local legislation and institutional requirements. Written informed consent to participate in this study was provided by the participants or participants’ legal guardian/next of kin.

## Author contributions

CZ: Writing – original draft, Writing – review & editing. JW: Data curation, Methodology, Writing – review & editing.
